# Response of alfalfa growth and rhizosphere properties to soil phosphorus supply and mowing in a salt-affected soil

**DOI:** 10.3389/fpls.2025.1565162

**Published:** 2025-05-06

**Authors:** Junjie Tian, Weifan Wan, Qian Liu, Bayinnamula Zhao, Haigang Li

**Affiliations:** ^1^ Inner Mongolia Key Laboratory of Soil Quality and Nutrient Resources, Inner Mongolia Agricultural University, Hohhot, China; ^2^ Key Laboratory of Agricultural Ecological Security and Green Development at Universities of Inner Mongolia Autonomous, Inner Mongolia Agricultural University, Hohhot, China

**Keywords:** alfalfa, phosphorus, mowing, carboxylate exudation, rhizosphere pH

## Abstract

**Introduction:**

Phosphorus (P) deficiency is a key limiting factor for alfalfa quality and yield in salt- affected soils, and root exudates are considered as a strategy for alfalfa P acquisition under P stress, which requires the supply of photosynthetic products.However, mowing may reduce the supply of photosynthetic products by decreasing photosynthetic rate and chlorophyll yield, which can affect the concentration of alfalfa root exudates in saline soil. This study aimed to investigate the possible mechanism of increasing P utilization rate with different P application rates after mowing.

**Methods:**

Field and pot experiments were conducted with alfalfa grown under five P application rates (0, 60, 120, 180 and 240 kg ha^-1^). Plant growth, shoot P concentration, rhizosphere carboxylates concentration, rhizosphere acid phosphatase (APase) activity and rhizosphere pH were measured.

**Results and discussion:**

P application significantly improved plant height and branch number, with the highest levels observed when P application rate was 180 kg ha^-1^. Crude protein content was also improved (13.3%-23.3%) with increasing P application rates and reached the maximum when P application rate was 240 kg ha^-1^. The yield of alfalfa increased with higher shoot P concentration. Furthermore, mowing significantly reduced rhizosphere carboxylates concentration (30.4%-100%) and APase activity (39.8%-75.0%) across all P treatments. However, these reductions were less pronounced when the P application rate exceeded 120 kg ha^-1^. In contrast, rhizosphere pH was unaffected by mowing across different application rates. Overall, this study demonstrates that P fertilization not only promotes alfalfa growth and quality in salt-affected soil but also mitigates the adverse effects of mowing on rhizosphere carboxylates concentration and APase activity. This highlights the potential of P fertilization to reduce P fixation and enhance P uptake by regulating rhizosphere-activated soil P dynamics after mowing. These findings provide a basis for enhancing the P utilization rate after mowing. The research results provide a basis for improving P utilization after mowing, enabling farmers to formulate scientific mowing strategies in production, thereby enhancing the yield and quality of alfalfa.

## 1 Introduction

Phosphorus (P) is one of the 17 essential elements required for plant growth (Raghothama and Karthikeyan, 2005). The concentration of P in plants typically ranges from 0.05% to 0.50% by dry weight. This vital element participates in a series of processes, including energy production, nucleic acid synthesis, photosynthesis, glycolysis, respiration, membrane synthesis and stability, enzyme activation, redox reactions, signal transduction, carbohydrate metabolism, and nitrogen (N) fixation (Plaxton and Tran, 2011). P deficiency is one of the most significant constraints on agricultural productivity worldwide (Richardson et al., 2011). This issue often arises not due to low total soil P but rather the limited availability of P in forms that plants can absorb (Simpson et al., 2011, 2015). A substantial portion of P applied to soils tends to bound to the surface of soil particles, or precipitates with calcium, aluminum, and iron, and hence P is relative poorly available to plants (Simpson et al., 2011). Orthophosphate, the primary form of P absorbed by plants, is typically present in soil solutions at very low concentrations (Hossner et al., 1973; Yanai, 1991; Vaz et al., 1993), well below the Orthophosphate concentration required for optimal growth of many crops (Fang et al., 2009).

Large amounts of P fertilizer have been applied to low P soils around the world to increase agricultural production (Townsend and Porder, 2012). However, only 15%-30% of P applied is absorbed by crops in the year of application (Syers et al., 2008). Thus, P fertilizer application often exceeds plant requirement, leading to the accumulation of large amounts of P in soils over time (Vu et al., 2008). In 2010, the application of P fertilizer in China is 8.06 million tons, that is, 6.05 million-7.25 million tons of P will remain in the soil, which is calculated according to the P accumulation rate (Cheng et al., 2010). This surplus contributes to serious environmental problems, such as the eutrophication of aquatic ecosystems (Carpenter, 2005). In addition, as a non-renewable resource, phosphate rock is consumed rapidly (Fixen and Johnston, 2012). To address these challenges, it is urgent to improve the efficiency of P acquisition and utilization in plants and to make sustainable use of P fertilizer (Johnston et al., 2014; Cordell and White, 2015).

Under low P conditions, plants typically prioritize the transport of photosynthates to their root systems to promote root growth and development in saline-alkali soils, thereby enhancing P uptake from the soil (Lynch et al., 2022). P-deficient plants enhance their ability of P acquisition through a series of physiological, biochemical and molecular adjustments, including carboxylates exudation (e.g., citrate, oxalate, and malate) and rhizosphere pH modification (Plaxton and Tran, 2011). The carboxylates mobilize sparingly soluble soil P by competing with both inorganic and organic P for binding sites in the soil (Lambers et al., 2017). The protons released into the rhizosphere by plants, can lead to rhizosphere acidification, thereby increasing P availability in alkaline soils. For instance, P availability is enhanced by rhizosphere acidification resulting from proton release during N_2_-fixation by some legumes (Hinsinger et al., 2003; Veloso et al., 2023). Increasing phosphatase secretion of roots, is another physiological strategy of P deficient plants to mobilize Orthophosphate (Nuruzzaman et al., 2006; Playsted et al., 2006). Jiang et al. (2003) found that approximately 60% of beans had increased the activity of APase in leaves under the treatment of no P supply. Similar findings were reported in a study of maize, that is, APase activity increased in low P treatment compared to high P treatment (Shuang et al., 2015). APase is mainly derived from plant secretion and it drives soil organic P hydrolysis, which is an adaptation strategy to low P stress (Hou et al., 2015).

Alfalfa (*Medicago sativa* L.) is an important Fabaceae perennial herb, which was widely cultivated across the world (Al-Kahtani et al., 2017). In China, alfalfa production is often limited by soil low P availability (He et al., 2017). When P supply is limited, both the yield and quality of alfalfa are decreased, nitrogen fixation is inhibited, and the number and size of root nodules are significantly reduced, leading to weakened nitrogen fixation (Pang et al., 2010). The application of P can increase alfalfa yield, quality, nodulation, and N fixation (Reid et al., 2006). P-deficient alfalfa roots acidify the rhizosphere to facilitate the dissolution of calcium phosphate in the soil and exude carboxylates such as malate, citrate, and succinate, which chelate iron and enhance P solubility, thereby improving P uptake (Richardson et al., 2011).

After defoliation, the growth of aboveground plant parts is constrained, and photosynthates are preferentially allocated to root system development under P-deficient conditions (Prescott et al., 2020, 2021). Some studies have shown that mowing directly reduces forage biomass, resulting in a decline in photosynthetic capacity, which in turn decreases the supply of photosynthetic products to the roots (Unkovich et al., 1997; Gao et al., 2008; Ilmarinen et al., 2008). Additionally, previous study demonstrated that defoliation decreases the concentration of rhizosphere carboxylates in sandy loam soil (Song et al., 2022). However, the effects of different P application rates on rhizosphere carboxylate concentrations, APase activity and rhizosphere pH in salt-affected soil after mowing were relatively unknown. In the present study, we conducted both field and pot experiments to evaluate the growth response of alfalfa under different P rates in a salt-affected soil. The primary objectives of this study were to investigate the effects of P application on alfalfa growth and to explore the possible mechanisms enhancing P acquisition in alfalfa under these conditions. We hypothesize that (i) mowing reduces the concentration of carboxylates and APase activity in rhizosphere; (ii) P application alleviates these reductions.

## 2 Materials and methods

### 2.1 Field experiment

#### 2.1.1 General situation of test site

The field experiment was set up during 2020-2022 at Hailiutu Modern Agriculture and Animal Husbandry Science and Technology Park (40°38’ N, 111°28’ E) of Inner Mongolia Agricultural University, Tumd Left Banner, Hohhot, Inner Mongolia Autonomous Region. This region is characterized by a temperate continental arid climate, with the annual maximum temperature occurring in July and the minimum temperature in January. Precipitation is unevenly distributed throughout the year, mainly concentrated from June to August. The site experiences an annual rainfall of 335.2–534.6 mm and an average temperature of 6.7°C. Prior to the experiment, the initial soil (calcareous alkaline soil) properties were as follows: pH (H_2_O), 8.42; soil organic matter, 17.44 g kg^-1^; NH_4_-N, 2.05 mg kg^-1^; NO_3_-N, 6.19 mg kg^-1^; Olsen-P, 9.99 mg kg^-1^, NH_4_OAc-K, 95.37 mg kg^-1^, salinity, 0.27 g kg^-1^, respectively.

#### 2.1.2 Experimental design and sampling

There were five P application rates in this experiment (kg P_2_O_5_ ha^-1^), including 0 (P0), 60 (P60), 120 (P120), 180 (P180) and 240 (P240), which covers a span from P-deficient to P-rich conditions, enabling the determination of optimal P application rates and providing guidance for agricultural practices. P fertilizer was supplied as superphosphate (P_2_O_5_ 18%). The amount of nitrogen fertilizer as urea (N 46%) and potassium fertilizer as sulfate (K_2_O 50.3%) were 90 kg ha^-1^ and 120 kg ha^-1^, respectively. All fertilizers were applied as basal dressing once annually in the spring and incorporated into the topsoil (0–20 cm) using a rotary tiller.

The experiment employed a randomized block design with three replicates per treatment, and each plot covered an area of 12 × 5 m². The alfalfa cultivar Zhongmu No. 1 was sown in 2020 at a seeding rate of 15 kg ha^-1^, with rows spacing 20 cm apart and a sowing depth of 1.2–2.0 cm. Plants were mowed at the early flowering stage on July 30, 2021 (the first year of planting), and June 30, 2022 (the second year of planting), respectively. Unmowed alfalfa was kept as control. Sickles were used to harvest three 10-cm-wide swaths in each plot.

Root samples were randomly taken from each plot with a root drill 7 cm in diameter. In the second year of planting, root samples were taken on the first, third, fifth, and ninth days after mowing. The root growth tended to be stable on the ninth day after mowing. By setting the sampling intervals, the key nodes of alfalfa regeneration dynamics were captured. Since root samples from the 0-20 cm layer can more sensitively reflect short-term responses of plant growth to mowing and P treatments, a 1.5 cm diameter auger was used to randomly collect 5 soil cores (0-20 cm) from each plot, which were subsequently combined into a composite sample for analysis.

#### 2.1.3 Plant growth measurements

Ten plants were randomly selected from each plot for growth assessments. Plant height was measured with a precision of 1 mm, and the number of branches on the main stem was recorded. Subsamples from each plot were weighed, dried at 65°C to constant weight and weighed again to determine alfalfa yield. The plant height, branch number, yield and the stem-leaf ratio of alfalfa were evaluated at the early flowering stage.

#### 2.1.4 Determination of shoot P concentration and soil Olsen-P

Dried shoot and root samples were ground to a fine powder, and approximately 0.15 g was digested in an H_2_SO_4_-H_2_O_2_ mixture. P concentration in plant tissue was determined by molybdenum-antimony spectrophotometric method (Westerman, 1990). Soil samples were dried and sieved at 2-mm, then determined Olsen P by NaHCO_3_ -P (Olsen et al., 1954).

#### 2.1.5 Determination of root exudates

Roots were gently shaken to remove bulk soil, and rhizosphere soil was defined as the soil adhering tightly to the root system. Roots were transferred to a cup filled with 50 mL 0.2 mM CaCl_2_ solution for extraction for 1 min. After taking out the root system, the extraction solution was separated three parts, which are respectively used to determine the APase activity, carboxylates concentration, and rhizosphere pH. Subsamples of the rhizosphere extract were kept in -20°C for analysis (Pang et al., 2010).

Carboxylates are quantified using Reversed-phase high performance liquid chromatograph (HPLC). Approximately 1 mL subsample of the rhizosphere extract was filtered into an HPLC vial. A 250 × 4.6 mm Altima C-18 reversed-phase chromatographic column was used for detection conditions. The mobile phase was 25 mmol L^-1^ KH_2_PO_4_ (with pH = 2.25) and the flow rate was 1.5 mL min^-1^. The column temperature was 37°C and the detection wavelength of UV detector (SPD-10A) was 214 nm (Cawthray, 2003).

APase activity was determined using *p*-nitrophenyl phosphate as a substrate, following the method of Tabatabai and Bremner (1969). The 0.1 mL of disodium *p*-nitrophenyl phosphate was added to the sample of soil suspension, prepared with a sodium acetate buffer. After 30 min incubation at 25°C, put the clear supernatant to a clean test tube and the 0.5 ml of 0.5 M NaOH was added to terminate the reaction. The absorbance of the filtrate was measured spectrophotometrically at 405 nm. The weight of the rhizosphere soil was calculated on a dry weight basis. APase activity was expressed as mmol *p*NP per gram of dry soil per hour (mmol *p*-NP g^−1^ h^−1^), with activity quantified using a standard curve.

The pH of rhizosphere extraction solution was measured. The experiment has indicated that the pH of rhizosphere is influenced by the amount of rhizosphere soil. The pH of rhizosphere was corrected according to the soil:water ratio of 1:5. The following equation was obtained from the experiment to correct rhizosphere pH: y= 0.1215ln(x) + 8.9988, R^2^ = 0.95, *P*<0.05, y, ratio of modified pH to original pH and, w: dry weight of the rhizosphere soil. The modified pH was regarded as rhizosphere pH.

### 2.2 Pot experiment

To verify the findings of field experiment regarding the effects of mowing on rhizosphere properties, a pot experiment was conducted with two P application rates and two treatments (mowing and control). P application rates were 0 and 200 mg P kg^–1^ as KH_2_PO_4_. When the application amount of P is 200 mg kg^-1^, it is equivalent to the application amount of 180-240 kg ha^-1^ in field. The objective of the pot experiment was to investigate the responses of alfalfa rhizosphere properties to mowing under both sufficient P application and no P application. Each treatment was replicated three times. The same soil and basal nutrients were used in this experiment. Soil was collected from the top layer (0–20 cm) of an undisturbed area in the field experiment site. After air dried, the soils were sieved at 2 mm. Basal nutrients were added into soil at the following rates (mg kg^–1^): urea 200, KH_2_PO_4_ 200, MgSO_4_·7H_2_O 43.34, CaCl_2_ 125.67, EDTA-Fe-Na 5.8, MnSO_4_·H_2_O 6.67, ZnSO_4_·7H_2_O 10, CuSO_4_·5H_2_O 2, H_3_BO_3_ 0.67, Na_2_MoO_4_·2H_2_O 0.26. The cultivar of alfalfa used in this experiment was the same as that in the field experiment. Soil moisture in pots were maintained at 70% field capacity by weighing. The seedlings in each pot (six per pot) were harvested at 52 days after sowing. Mowed at the early flowering stage. Sampling was performed on the first, third, fifth, ninth and fourteenth days after mowing. The same treatment alfalfa without mowing was grown as control. The concentration of carboxylates in rhizosphere, APase activity and rhizosphere pH were measured using the method as field experiment.

### 2.3 Data analyses

A one-way analysis of variance using the SAS statistical software (SAS 2001, SAS institute Inc., USA). Significant differences among means were determined by LSD at the 0.05 probability treatment. Normal distribution was tested for data using SPSS (SPSS Inc., Chicago, IL, USA). All figures were generated using Origin software (Origin 2021, OriginLab, USA).

## 3 Results

### 3.1 Alfalfa growth

**Figure 1:**
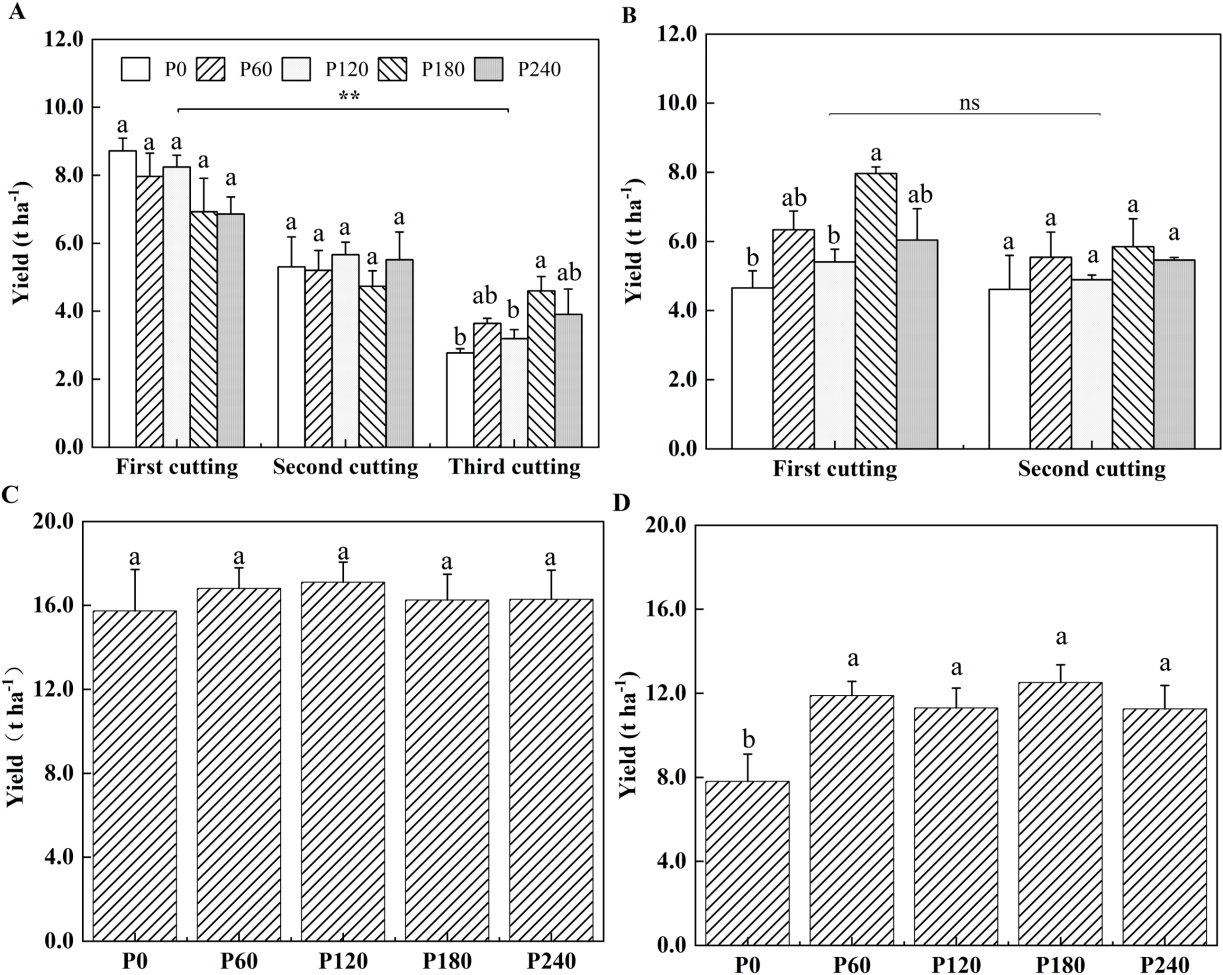
Alfalfa yield per cutting in 2021 **(A)** and 2022 **(B)**. Total alfalfa yield in 2021 **(C)** and 2022 **(D)**. Each value is the mean (+SE) of three replicates. Different letters denote significant differences among P application rates (*P*<0.05). ** indicate significant differences among cuttings (*P*<0.01). ns indicates that there is no significant difference among cuttings.

Alfalfa yield ranged between 4.56 t ha^−1^ and 8.72 t ha^−1^ with no significant differences observed among five P application rates at the first and second cuttings in the first year of planting (**Figure 1A**). Compared with P0, alfalfa yield significantly increased by 39% in P180 with the same plant height but a higher branches number at the third cutting in the first year of planting (**Figure 1A**, **Supplementary Figures S1A, S2A**). Plant height showed the highest in P240, but it cannot cause higher yield in the first year of planting (**Supplementary Figure S1A**). At the second year of planting, alfalfa yield in P180 was also the highest at the first cutting, which was significantly higher by 42% than that in P0, while no significant differences were observed at the second cutting among treatments (**Figure 1B**). However, P fertilization did not change plant height and branch number in all the treatments (**Supplementary Figures S1B, S2B**). Total alfalfa yield did not respond to P application in the first year (**Figure 1C**), but in the second year, P fertilization significantly increased total yield by 44.9% to 60.3% compared to P0 (**Figure 1D**). In the pot experiment, compared to P0, alfalfa shoot biomass significantly increased by 71.4% in P200 treatment with a higher plant height and branch number for 52 days growth period (**Supplementary Figures S3A, B, S4**).

### 3.2 Alfalfa quality

**Figure 2:**
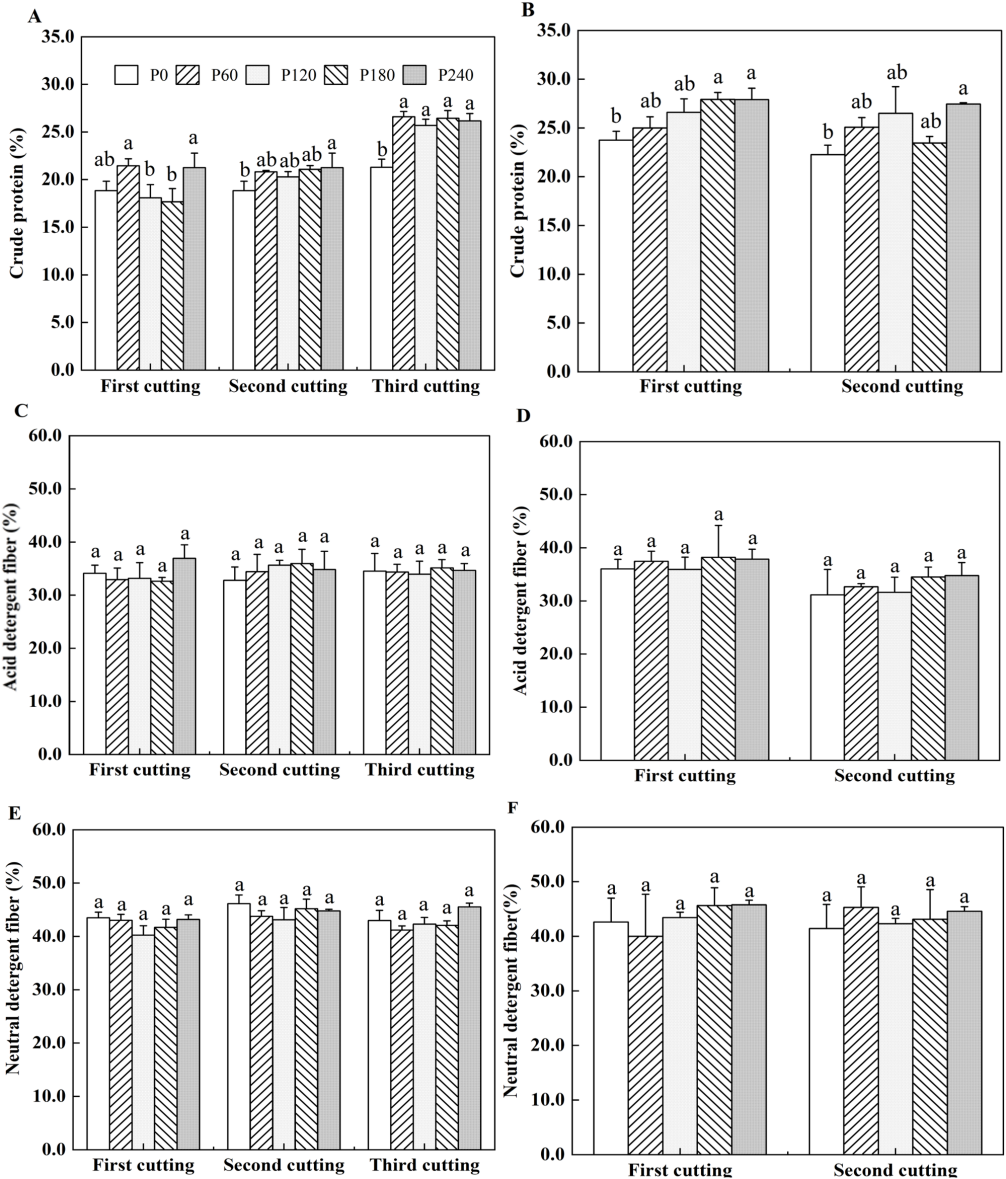
Alfalfa crude protein in 2021 **(A)** and 2022 **(B)**. Acid detergent fiber (ADF) of alfalfa in 2021 **(C)** and 2022 **(D)**. Neutral detergent fiber (NDF) of alfalfa in 2021 **(E)** and 2022 **(F)**. Each value is the mean (+SE) of three replicates. Different letters denote significant differences (*P*<0.05).

In the first year of planting, compared with P0, the crude protein content of alfalfa was significantly increased by 13.3%, 17.4% and 22.0% in P240 treatment at the first, second and third cuttings, respectively. In the second year of planting, crude protein content was also affected by P level, as they were 17.7% higher in P240 than that in P0 at the first cutting (**Figure 2A**). Crude protein content was significantly higher by 23.3% in P240 compared to P0 at the second cutting (**Figures 2A, B**). The acid detergent fiber content of alfalfa ranged from 32.6% to 36.9% in the first year of planting and from 31.1% to 38.2% in the second year of planting with no significant differences observed among different P application rates (**Figures 2C, D**). Same to acid detergent fiber content, neutral detergent fiber content of alfalfa did not show significant variation across different P application rates, ranging from 40.3% to 46.2% in the first year of planting and from 40.0% to 45.8% in the second year of planting (**Figures 2E, F**). The stem/leaf ratio was between 0.95 and 1.8 in the first year of planting and between 0.93 and 1.46 in the second year of planting with no significant differences observed among different treatments (**Supplementary Figures S5A, B**).

### 3.3 Shoot P concentration

**Figure 3:**
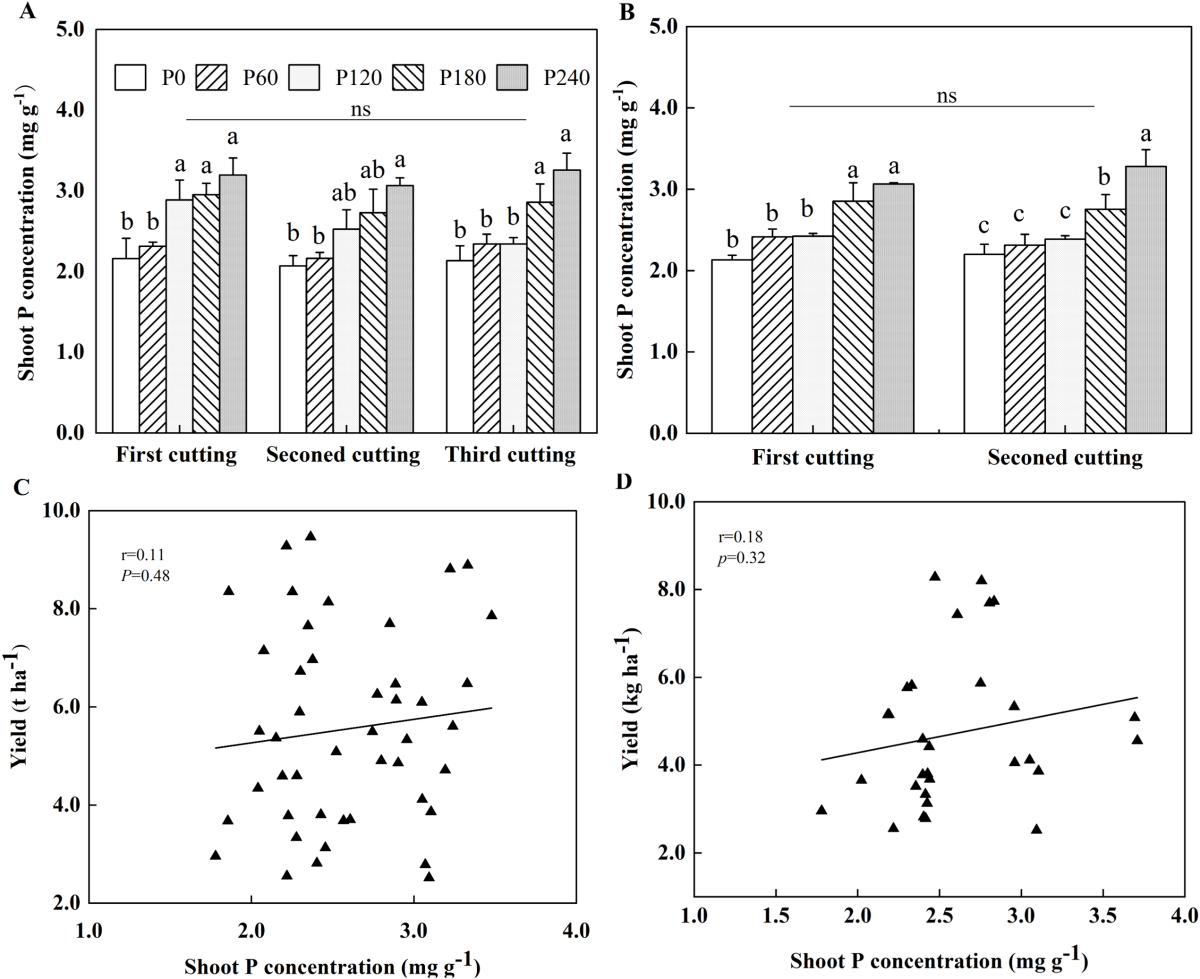
Alfalfa shoot P concentration in 2021 **(A)** and 2022 **(B)**. Each value is the mean (+SE) of three replicates. Different letters denote significant differences (*P*<0.05). ns indicates that there is no significant difference among cuttings. Correlation between shoot P concentration and alfalfa yield in 2021 **(C)** and 2022 **(D)**. Data point represents individual replicate.

P application significantly increased shoot P concentration of alfalfa grown in both years of the field experiment (**Figures 3A, B**). Specifically, compared to P0, shoot P concentration of alfalfa was elevated by 25%-32% in high P treatments, such as P120, P180 and P240 at the first cutting in the first year of planting, with a 32% increase observed in P240 at the second cutting. At the third cutting, the P application further enhanced shoot P concentration, with the greatest increases of 26% and 34% under P180 and P240, respectively (**Figure 3A**). In the second year of planting, shoot P concentration under P180 and P240 were significantly 25% and 35% higher than P0 at the first cutting, and were 33% higher in P240 than P0 at the second cutting (**Figure 3B**). No significant correlation was found between alfalfa yield and shoot P concentration across both years (**Figures 3C, D**). In pot experiment, P addition cannot cause a higher P concentration, and mowing did not affect the shoot P concentration (**Supplementary Figure S6**).

### 3.4 Soil Olsen-P

**Figure 4:**
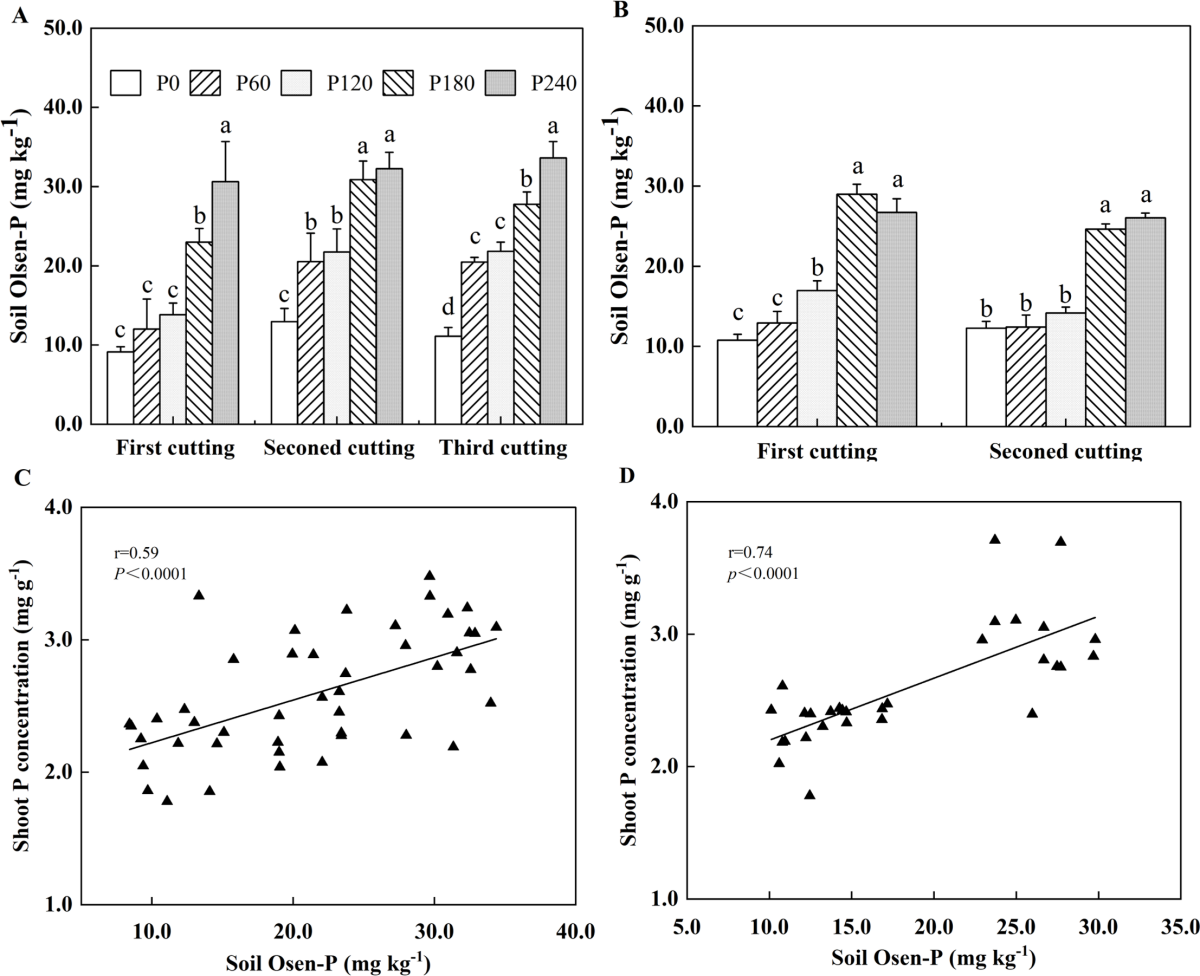
Soil Olsen-P in 2021**(A)** and 2022 **(B)**. Each value is the mean (+SE) of three replicates. Different letters denote significant differences (*P*<0.05), Correlation between shoot P concentration and soil Olsen-P in 2021 **(C)** and 2022 **(D)**. Data point represents individual replicate.

P application significantly increased soil Olsen-P only when its rates were 180 and 240 kg ha^-1^ at the first cutting (**Figure 4A**). In contrast, a significant increase in soil Olsen-P was observed at all P application rates, with an increase of 59%-67% at the second and third cuttings in the first year of planting. In the second year of planting, the relative low P rates, such as 60 mg kg^-1^ at the first cutting and 60-120 mg kg^-1^ at the second cutting, did not significantly affect soil Olsen-P compared to P0 (**Figure 4B**). As P application increased to 240 kg ha^-1^, the maximal soil Olsen-P concentrations reached 33.61 mg kg^−1^ in the first year of planting and 28.98 mg kg^−1^ in the second year of planting. Pearson’s correlation analysis revealed a positive relationship between shoot P concentration and soil Olsen-P (**Figures 4C, D**).

### 3.5 Carboxylates concentration in rhizosphere

In the second year of planting in field experiment, the response of carboxylate concentrations in rhizosphere of alfalfa to P application was pronounced. Malate was present in the largest amounts in exudates of alfalfa in P0 compared with P addition. The concentrations of citrate, succinate and tartrate reached the highest at a P application rate of 60 kg ha^-1^. Tartrate constituted the smallest proportion of the carboxylates in the root exudates.

The carboxylates concentrations in rhizosphere of alfalfa can be inhibited by mowing within three days in the field (**Table 1**). Specifically, malate concentration in rhizosphere in control (Con, no mowing) was significantly higher than that in mowing treatments in P120, P180 and P240 (the first day after mowing). However, no significant differences in malate concentrations were observed on the third, fifth, and ninth days following mowing. Similarly, citrate concentrations in mowing treatment were significantly lower than that of control in P120, P180, P240 on the first and third days after mowing. By the fifth and ninth days, no significant differences in citrate concentrations were observed between mowing and control treatments across all P application rates. Mowing increased succinate concentration in alfalfa rhizosphere to the maximum of 4.31 μmol g^−1^ soil in P0, which was four times greater than control on the first day after mowing. However, on the third, fifth, and ninth days after mowing, the succinate concentrations had no significant difference between mowing and control treatments among five P application rates. In the case of tartrate, mowing resulted in a 72.0% and 78.4% reduction in concentrations in the P180 and P240 treatments, respectively, compared to the control on the first day after mowing. Similar to succinate, no significant differences in tartrate concentrations were found between mowing and control treatments on the third, fifth, and ninth days after mowing across all P application rates.

**Table 1:** Amounts of carboxylates in rhizosphere of alfalfa at the first, third, fifth and ninth days after mowing and non-mowing in field experiment in 2022.

Concentrations of carboxylates in rhizosphere (μmol g^-1^ soil)									
Carboxylates	Phosphorus rate (kg ha ^-1^)	1 day		3 day		5 day		9 day	
		Control	Mowing	Control	Mowing	Control	Mowing	Control	Mowing
Malate	0	2.36 ± 1.28a	2.37 ± 0.65a	1.39 ± 0.21a	1.01 ± 0.05a	4.11 ± 0.85a	4.31 ± 1.03a	2.61 ± 0.68a	2.16 ± 0.41a
	60	1.36 ± 0.68a	1.23 ± 0.03a	1.26 ± 0.37a	0.97 ± 0.08a	1.80 ± 0.47b	3.68 ± 0.46a	1.97 ± 0.14a	1.80 ± 0.80a
	120	1.57 ± 0.17a	0.42 ± 0.24b	1.01 ± 0.26a	0.41 ± 0.22a	2.12 ± 0.69a	1.88 ± 0.38a	1.93 ± 0.46a	1.21 ± 0.25b
	180	1.61 ± 0.18a	0.46 ± 0.17b	0.56 ± 0.13a	0.38 ± 0.03a	2.99 ± 0.65a	2.75 ± 0.09a	1.63 ± 0.42a	1.67 ± 0.25a
	240	1.36 ± 0.19a	0.36 ± 0.12b	0.39 ± 0.21a	0.50 ± 0.25a	1.80 ± 0.12a	2.03 ± 0.27a	1.36 ± 0.06a	1.83 ± 0.70a
Citrate	0	2.26 ± 0.34a	1.42 ± 0.48b	2.41 ± 0.56a	3.08 ± 0.64a	2.46 ± 0.57a	3.46 ± 0.27a	2.62 ± 0.85a	2.71 ± 0.30a
	60	3.76 ± 0.40a	1.84 ± 0.06b	1.44 ± 0.02a	1.02 ± 0.24a	2.74 ± 0.23a	3.15 ± 0.54a	2.02 ± 0.73a	2.41 ± 0.20a
	120	3.65 ± 0.06a	2.68 ± 0.54a	4.04 ± 0.68a	1.05 ± 0.35b	3.69 ± 0.66a	3.03 ± 0.36a	1.67 ± 0.26a	2.56 ± 0.26a
	180	2.51 ± 0.93a	1.29 ± 0.77a	2.24 ± 0.97a	0.55 ± 0.28b	3.35 ± 0.92a	3.67 ± 0.56a	1.55 ± 0.41a	1.81 ± 0.34a
	240	2.37 ± 0.51a	1.38 ± 0.41a	1.88 ± 0.70a	0.70 ± 0.31a	2.83 ± 0.39a	2.69 ± 0.42a	1.57 ± 0.52a	2.39 ± 0.78a
Succinate	0	0.95 ± 0.72b	4.31 ± 0.63a	1.72 ± 0.47a	1.47 ± 0.30a	0.95 ± 0.10b	2.08 ± 0.69a	1.25 0.245a	1.34 ± 0.28a
	60	2.05 ± 0.22a	3.22 ± 0.72a	1.01 ± 0.08b	1.80 ± 0.11a	1.02 ± 0.28b	3.62 ± 0.07a	2.46 ± 0.38a	1.91 ± 0.96a
	120	2.30 ± 0.51a	3.01 ± 0.93a	2.04 ± 0.74a	1.40 ± 0.13a	1.78 ± 0.31a	1.40 ± 0.47a	3.32 ± 0.33a	3.48 ± 0.90a
	180	2.47 ± 0.42a	2.51 ± 0.55a	1.84 ± 0.45a	0.87 ± 0.24a	1.09 ± 0.25a	1.27 ± 0.77a	2.31 ± 0.09a	2.13 ± 0.20a
	240	0.98 ± 0.18a	2.80 ± 0.26b	1.62 ± 0.20a	1.07 ± 0.54a	1.08 ± 0.16a	1.03 ± 0.18a	1.32 ± 0.43a	1.04 ± 0.58a
Tartrate	0	1.51 ± 0.56a	0.71 ± 0.16a	0.78 ± 0.14a	0.69 ± 0.28a	0.63 ± 0.18a	0.83 ± 0.10a	0.61 ± 0.17a	0.29 ± 0.02a
	60	1.36 ± 0.42a	1.51 ± 0.01a	0.76 ± 0.13a	1.43 ± 0.23a	0.45 ± 0.11a	1.67 ± 0.23b	0.72 ± 0.44a	0.44 ± 0.11a
	120	2.22 ± 0.08a	1.32 ± 0.56a	1.83 ± 0.02a	0.57 ± 0.04b	0.52 ± 0.14a	0.54 ± 0.11a	1.49 ± 0.29a	0.25 ± 0.07b
	180	2.07 ± 0.27a	0.57 ± 0.34b	1.06 ± 0.20a	0.26 ± 0.13a	0.52 ± 0.14a	0.52 ± 0.22a	0.56 ± 0.08a	0.54 ± 0.06a
	240	1.67 ± 0.27a	0.36 ± 0.02b	0.75 ± 0.04a	0.26 ± 0.13b	0.87 ± 0.47a	0.32 ± 0.04a	0.68 ± 0.32a	0.20 ± 0.06a

Each value is the mean (± SE) of three replicates. Different letters denote significant differences between mowing and control (P<0.05).

In pot experiment, the concentration of carboxylates in rhizosphere was lower in P200 compared to P0. Mowing had negative effects on the secretion of carboxylates (**Table 2**). For P0 treatment, mowing decreased the concentrations of carboxylates. Specifically, when compared to control, malate concentrations significantly reduced by 63.6%-100% from the first day to the fourteenth day after mowing. Citrate concentrations were also significantly decreased by 57.1%-91.7% from the third day to the fourteenth day after mowing, compared to control. Similarly, tartrate concentrations declined by 30.4%-94.7% from the first day to the ninth day after mowing compared to the control. A similar pattern was observed in the P200 treatment, compared to control, mowing significantly reduced the concentrations of malate, citrate and tartrate by 86.6%-100%, 57.1%-89.5% and 63.0%-100% from the first day after mowing to the fourteenth day after mowing, respectively.

**Table 2:** Carboxylates concentration in rhizosphere of alfalfa at the first, third, fifth, ninth and fourteenth days after mowing and non-mowing in pot experiment.

Concentration of carboxylates in rhizosphere (μmol g^-1^ soil)											
Carboxylates	Phosphorus rate (mg kg^-1^)	1 day		3 day		5 day		9 day		14 day	
		Control	Mowing	Control	Mowing	Control	Mowing	Control	Mowing	Control	Mowing
Malate	0	0.07 ± 0.02a	0.02 ± 0.01b	0.05 ± 0.02a	1.01 ± 0.05b	0.11 ± 0.02a	0.04 ± 0.01b	0.50 ± 0.07a	0.01 ± 0.00b	0.08 ± 0.01a	0b
	200	0.03 ± 0.01a	0.01 ± 0.00b	0.02 ± 0.00a	0.00 ± 0.00a	0.02 ± 0.00a	0.01 ± 0.00a	0.15 ± 0.04a	0.02 ± 0.00b	0.03 ± 0.01a	0b
Citrate	0	0.01 ± 0.00a	0.03 ± 0.01a	0.07 ± 0.02a	0.03 ± 0.01b	0.08 ± 0.01a	0.04 ± 0.01b	0.36 ± 0.05a	0.03 ± 0.01b	0.04 ± 0.00a	0.01 ± 0.00b
	200	0.04 ± 0.01a	0.01 ± 0.00b	0.05 ± 0.01a	0.01 ± 0.00b	0.07 ± 0.01a	0.03 ± 0.01b	0.19 ± 0.05a	0.02 ± 0.01b	0.07 ± 0.03a	0.01 ± 0.00b
Tartrate	0	0.36 ± 0.09a	0.03 ± 0.00b	0.300.04a	0.69 ± 0.28b	0.57 ± 0.05a	0.83 ± 0.10b	0.23 ± 0.01a	0.29 ± 0.02b	0.04 ± 0.01a	0a
	200	0.28 ± 0.05a	0.04 ± 0.01b	0.19 ± 0.03a	1.43 ± 0.23b	0.14 ± 0.02a	1.67 ± 0.23b	0.27 ± 0.05a	0.44 ± 0.11b	0.03 ± 0.01a	0b

Each value is the mean (± SD) of three replicates. Different letters denote significant differences between mowing and control (*P*<0.05).

### 3.6 APase activity and rhizosphere pH

In the second year of planting (**Table 3**) in field experiment, the APase activity was significantly reduced by 80.4% and 82.6% on the first day after mowing in P180 and P240 compared to control. On the third day after mowing, APase activity decreased by 63.2%, 68.8% and 64.9% in P60, P180 and P240 treatments, respectively, compared to control. By the ninth day after mowing, the activity decreased by 62% and 54% in the P60 and P120 treatments, respectively, compared to the control. On the fifth day after mowing, no significant differences in APase activity were observed among the five P application rates. In contrast, rhizosphere pH did not differ significantly between the mowing and control treatments.

**Table 3:** Acid phosphatase activity and rhizosphere pH of alfalfa at the first, third, fifth and ninth days after mowing and non-mowing in field experiment in 2022.

Indicator	Acid phosphatase activity (mg PNP g^-1^ soil h^-1^)								
	Phosphorus rate (kg ha ^-1^)	1 day		3 day		5 day		9 day	
		Control	Mowing	Control	Mowing	Control	Mowing	Control	Mowing
Acid phosphatase activity (mg PNP g^-1^ soil h^-1^)	0	1.81 ± 0.35a	1.28 ± 0.26a	2.19 ± 0.89a	4.28 ± 0.86a	1.70 ± 0.61a	2.33 ± 0.24a	7.40 ± 1.95a	4.61 ± 1.25a
	60	1.40 ± 0.36a	1.30 ± 0.65a	5.22 ± 1.16a	1.92 ± 0.52b	1.92 ± 0.44a	1.40 ± 0.53a	3.04 ± 0.15a	1.17 ± 0.37b
	120	1.63 ± 0.31a	2.00 ± 0.05a	2.62 ± 0.05a	4.67 ± 0.89a	3.10 ± 0.95a	2.28 ± 052a	3.67 ± 0.18a	1.69 ± 0.45b
	180	4.50 ± 0.39a	0.88 ± 0.30b	4.01 ± 0.53a	1.25 ± 0.32b	1.76 ± 0.09a	1.42 ± 0.01b	2.66 ± 1.44a	3.10 ± 0.75a
	240	3.57 ± 0.84a	0.62 ± 0.21b	2.28 ± 0.06a	0.80 ± 0.28b	4.70 ± 0.40a	2.68 ± 0.14a	3.02 ± 1.32a	1.12 ± 0.17a
Rhizosphere pH	0	7.23 ± 0.18a	6.82 ± 0.19a	6.58 ± 0.14a	6.74 ± 0.05a	6.02 ± 0.31a	6.37 ± 0.09a	6.14 ± 0.14a	6.37 ± 0.18a
	60	7.06 ± 036a	6.86 ± 0.11a	6.58 ± 0.06a	6.86 ± 0.08a	6.32 ± 0.06a	6.86 ± 0.17a	6.59 ± 0.10a	6.48 ± 0.16a
	120	7.08 ± 0.32a	6.94 ± 0.20a	6.75 ± 0.31a	6.57 ± 0.09a	6.31 ± 0.17a	6.63 ± 007a	6.54 ± 0.06a	6.55 ± 0.13a
	180	7.01 ± 0.20a	6.93 ± 0.20a	6.65 ± 0.22a	6.68 ± 0.10a	6.29 ± 0.14a	6.58 ± 0.02a	6.54 ± 0.15a	6.59 ± 0.09a
	240	6.90 ± 0.12a	6.66 ± 0.13a	6.65 ± 0.16a	6.90 ± 0.06b	6.31 ± 0.20a	6.28 ± 0.13a	6.37 ± 0.02a	6.24 ± 0.09a

Each value is the mean (± SE) of three replicates. Different letters denote significant differences between mowing and control (*P*<0.05).

In pot experiment, mowing had a negative effect on the APase activity (**Table 4**). In P0 treatment, compared to control, the APase activity in rhizosphere of alfalfa was unaffected on the first day of mowing, but it significantly decreased by 61.6%, 39.8%, 73.5%, 49.8%, on the third, fifth, ninth and fourteenth days after mowing, compared to control, respectively. In P200 treatment, when compared to control, the APase activity in rhizosphere of alfalfa was lower under mowing than that of control from the first day to the ninth day after mowing. Specifically, compared to control, the APase activity in rhizosphere significantly decreased by 75.0%, 60.6%, 70.0% and 59.1%, on the first, third, ninth and fourteenth days respectively. Like the field experiment, mowing did not significantly affect rhizosphere pH.

**Table 4:** Acid phosphatase activity and rhizosphere pH in rhizosphere of alfalfa at the first, third, fifth, ninth and fourteenth days after mowing and non-mowing in pot experiment.

Indicator		Phosphorus rate (mg kg^-1^)	1 day		3 day		5 day		9 day		14 day	
			Control	Mowing	Control	Mowing	Control	Mowing	Control	Mowing	Control	Mowing
Acid phosphatase activity (mg PNP g^-1^ soil h^-1^)		0	0.25 ± 0.05a	0.22 ± 0.01a	0.58 ± 0.07a	0.22 ± 0.06b	0.29 ± 0.04a	0.18 ± 0.02b	0.35 ± 0.02a	0.09 ± 0.02b	0.22 ± 0.02a	0.11 ± 0.01b
		200	0.40 ± 0.02a	0.10 ± 0.01b	0.33 ± 0.06a	0.13 ± 0.02b	0.19 ± 0.03a	0.14 ± 0.02a	0.30 ± 0.06a	0.09 ± 0.03b	0.22 ± 0.05a	0.09 ± 0.01b
Rhizosphere pH		0	7.39 ± 0.10a	7.63 ± 0.06a	8.08 ± 0.15a	7.64 ± 0.27b	8.22 ± 0.31a	8.37 ± 0.42b	8.23 ± 0.25a	8.06 ± 0.17b	7.96 ± 0.11a	7.83 ± 0.06b
		200	7.67 ± 0.10a	7.40 ± 0.04b	8.22 ± 0.31a	7.50 ± 0.22b	8.07 ± 0.17a	7.88 ± 0.13a	7.84 ± 0.11a	7.50 ± 0.16b	8.37 ± 0.36a	8.22 ± 0.15b

Each value is the mean (± SD) of three replicates. Different letters denote significant differences between mowing and control (*P*<0.05).

## 4 Discussion

### 4.1 Alfalfa growth, quality and P concentration

As the central component of ATP molecules, P enhances ATP synthesis efficiency, fueling cellular processes such as division and elongation to stimulate alfalfa plant height and branching (Plaxton and Tran, 2011). P fertilizer has been shown to increase alfalfa yield and promote plant growth (Larson et al., 1952). Suzuki (1991) reported that alfalfa yield was positively correlated with plant height and branch number. In the present study, P application significantly boosted plant height and branch number. Similar results have been reported for green manure crops (Elser et al., 2007; Peters and Stritzke, 1970). Specifically, P supply led to a notable increase in total alfalfa yield with the maximal value of 12.5 t ha^−1^ in P180 treatment in the second year of planting, though no such effect was observed in the first year (**Figures 1C, D**). As plants mature, competition among individuals intensifies, reducing available growth space and limiting nutrient uptake (Dai et al., 2017), which may explain why the increase in plant height and branch number in the first years did not correspond to a higher yield. Since P is relatively immobile in soil, its residual effects persist over time (Read and Winkleman, 1982), suggesting that the impact of P fertilization on alfalfa yield becomes more pronounced with prolonged application. Previous study has shown that P application significantly enhances the yield of maize and wheat (Xiu et al., 2012), further supporting the vital role of P in alfalfa growth and development. Mowing has been shown to reduce grass density, soil water content, and respiration rates, all of which can lead to decreased shoot biomass (Groffman et al., 1996). Consistent with these findings, our results indicated that with increasing cutting frequency, the yield of alfalfa per cutting significantly decreased, a trend also observed by Marshall et al. (2010).

Previous study has shown that P fertilizer increases crude protein content by enhancing nodule formation, which in turn promotes symbiotic nitrogen fixation (Sanderson and Jones, 1993). In the present study, alfalfa crude protein content was higher in P-supplied treatments compared to those without P, supporting the findings of Walworth and Summer (1990). Additionally, P application significantly increased shoot P concentration in alfalfa, consistent with Larson et al. (1952) and Pang et al. (2010), who observed that higher P application rates lead to increased plant P concentration. These results suggest that the positive effects of P fertilization on crude protein content and shoot P concentration contribute to enhanced alfalfa growth and yield. Farmers can apply P fertilizer at a rate of 180 kg ha^-1^ when planting alfalfa in saline-alkali soil to achieve optimal yield and quality.

### 4.2 Carboxylates concentration in rhizosphere

We hypothesized that mowing would decrease the concentration of carboxylates in the rhizosphere of alfalfa, regardless of P treatment. This hypothesis was fully supported. Both pot and field experiments demonstrated a significant reduction in rhizosphere carboxylate concentrations following mowing across all P application rates. The concentrations of the carboxylates were reduced due to the use of photosynthetic products for the regrowth of plants, leading to a substantial reduction in rhizosphere carboxylate concentrations post-mowing. This finding was consistent with previous studies in which the concentration of carboxylates in rhizosphere of L*. chinensis* was decreased by grazing (Song et al., 2022; Strom et al., 2001). Additionally, Bienvenu et al. (2022) also observed a slight reduction in the secretion ability of Asian flat root exudates by 18.7%–28.5% due to mowing. Prior research indicates that plant wounding causes a brief disruption in root exudation as stored compounds are mobilized during remobilization (Paterson and Sim, 1999; Danckwerts and Gordon, 1987). Similarly, root exudates of *Phleum pratense* (Mikola and Kytöviita, 2002) and maize (Nguyen and Henry, 2002) were reduced following leaf fall. There’s a reasonable explanation is that grazing is a factor that reduces the rate of photosynthesis and the total plant photosynthesis due to smaller leaf surface area, resulting in a reduced supply of photosynthetic products to roots (Zheng et al., 2017). After defoliation, both the underground biomass and main root diameter of alfalfa were decreased, and the growth of above-ground parts consumes energy stored in the roots (Tälle et al., 2018). Energy is primarily allocated to maintaining root respiration after defoliation, with only limited stored assimilates available for alfalfa growth (Bokhari and Singh, 1974; Schmitt et al., 2013), thereby reducing the concentration of carboxylates. Consequently, the photosynthates that support alfalfa growth also limit the intensity of rhizosphere processes, explaining the reduced carboxylate concentrations in the rhizosphere following mowing.

Our second hypothesis posited that P addition would alleviate the reduction in carboxylate exudation following mowing, and this hypothesis was supported. In high P treatments, the decrease in carboxylates concentrations in the alfalfa rhizosphere was less pronounced compared to treatments without P fertilization after mowing. This suggests that the negative effects of mowing on carboxylates concentrations can be mitigated by P application. Fertilization has been shown to enhance compensatory growth and improve the plant’s regenerative capacity (Mcnaughton et al., 1998). Previous study has indicated that mowing negatively impacts the growth of perennial herbs, but fertilization can promote compensatory growth following mowing (Hicks, 2000). Staalduinen and Peco (2010) conducted a greenhouse experiment on perennial grasses in Mediterranean and Central Asian steppes to test the importance of nutrient availability for compensatory growth after mowing and found that plants under high fertilization treatments were able to compensate for some of the potential losses caused by defoliation, with altered leaf allocation patterns. In high P treatments, it is likely that the regeneration of above-ground plant parts is enhanced, allowing more resources to be allocated to the underground parts, which in turn supports increased carboxylate secretion in the rhizosphere (Guo et al., 2012). However, the compensatory effect of fertilization on alfalfa is delayed, indicating that the benefit of fertilization is not immediate and may be limited over time (Reich et al., 1998).

Carboxylates with three carboxyl groups, such as citrate, are more effective at mobilizing P from soil particles than carboxylates with two carboxyl groups, such as malate, oxalate, and malonate (Mimmo et al., 2011). For instance, grazing has been found that increased the oxalate concentrations in rhizosphere of L*. chinensis* (Song et al., 2022). In the present study, succinate concentrations in alfalfa rhizosphere were higher than that in the control on the first day and third day after mowing in field experiment, which was in accordance with previous studies (He et al., 2021). Succinate may mobilize P as effectively as citrate but requires less carbon, which may explain the increased concentration of succinate in the low P treatment on the first day after mowing.

Jones (1998) reported that different growth stages and light conditions influence carboxylate concentrations, and Badri et al. (2014) observed significant changes in the root exudate composition of *Arabidopsis* during different growth and development stages. In our study, no significant differences in rhizosphere carboxylate concentrations were observed among the five P application rates in the field experiment on the fifth and ninth days after mowing. This lack of variation may be attributed to differences in the plant growth and developmental stages at these time points.

### 4.3 APase activity and pH in rhizosphere

APase activity in rhizosphere soil mainly originates from plant root exudates and soil microorganisms (Li et al., 2024). Previous study has shown that mowing significantly affects plant resource allocation, which in turn impacts the exchange of substances within plants and leads to a decrease in enzyme activity (Kandeler et al., 2006). Previous study has shown that microbial abundance is positively correlated with enzyme activity (Wallenius et al., 2011). As a highly active component of soil organic matter, soil microbial biomass can be reduced by mowing, which in turn leads to a decrease in enzyme activity (Li et al., 2014). It has been found that the activity of alkaline phosphatase decreased by 28.9%-44.2% after mowing (Qin et al., 2016). A similar reduction in enzyme activity was observed in grazed pasture soils, where enzyme activity was significantly lower compared to non-grazed soils (Cui and Holden, 2015). In the present study, the APase activity in rhizosphere of alfalfa was lower after mowing than in the control treatments both in the field and pot experiments. This decrease in activity may be attributed to the energy required for plant growth and development, which limits the secretion of APase by alfalfa roots, consistent with previous findings (Ping, 2002).

Soil pH is a key factor determining the availability of P (Lambers et al., 2008). It has been found that rhizosphere pH was increased by grazing (Song et al., 2022). Our results did not support this observation. Rhizosphere pH did not differ significantly between treatments across different P application rates. This may be explained by mowing reducing carboxylate concentrations in the alfalfa rhizosphere, which in turn limits any potential changes in pH (Nye, 1972, 1981).

## 5 Conclusions

Alfalfa growth and shoot P concentration were the greatest when the amount of P application was 180 kg ha-1. Mowing decreased rhizosphere carboxylates concentration and APase activity, while rhizosphere pH remained unaffected. The rhizosphere carboxylates concentrations and APase activity were least reduced by mowing when P application rate was higher than 120 kg ha-1. Additionally, P addition alleviated the reduction of carboxylate exudations after mowing. This study provides valuable insights into improving the utilization rate of P fertilizer after mowing. In the future, it may be possible to explore the interactive mechanisms between defoliation-induced carbon allocation strategies and rhizosphere microbial community functions, and to unravel the signaling roles of root exudates under combined salinity and phosphorus deficiency stresses.

## Data Availability

The original contributions presented in the study are included in the article/**Supplementary Material**, further inquiries can be directed to the corresponding author.
