# Supplementary material for: Response of alfalfa growth and rhizosphere properties to soil phosphorus supply and mowing in a salt-affected soil
Source: Front Plant Sci. 2025 May 6;16:1565162. doi: 10.3389/fpls.2025.1565162 (PMC12089102; doi:10.3389/fpls.2025.1565162)
Supplement: Supplementary file 1 [file DataSheet1.docx]

***Supplementary Material***


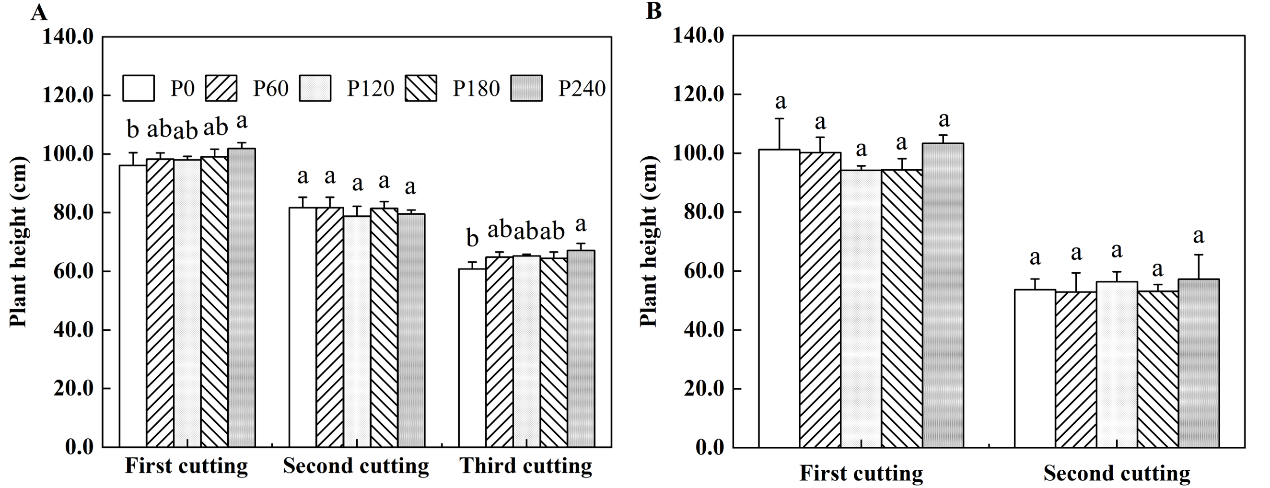


**Figure S1**. Alfalfa height in field experiment in 2021 **(A)** and 2022 **(B).** Each value is the mean (+SE) of three replicates. Different letters denote significant differences (*P*<0.05).


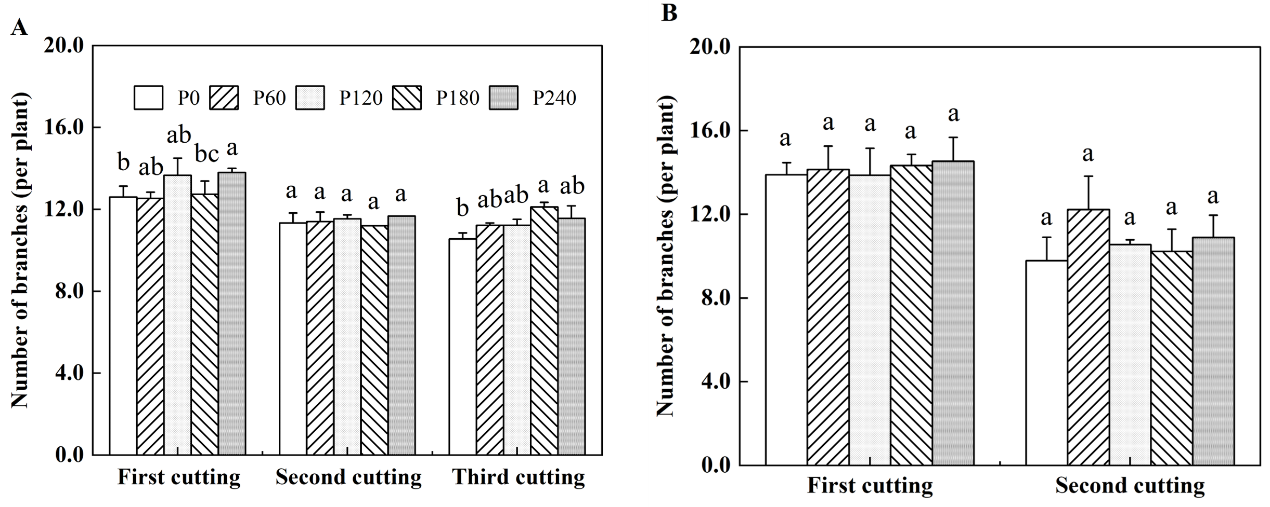


**Figure** **S2**. Alfalfa branch number in field experiment in 2021 **(A)** and 2022 **(B)**. Each value is the mean (+SE) of three replicates. Different letters denote significant differences (*P*<0.05).


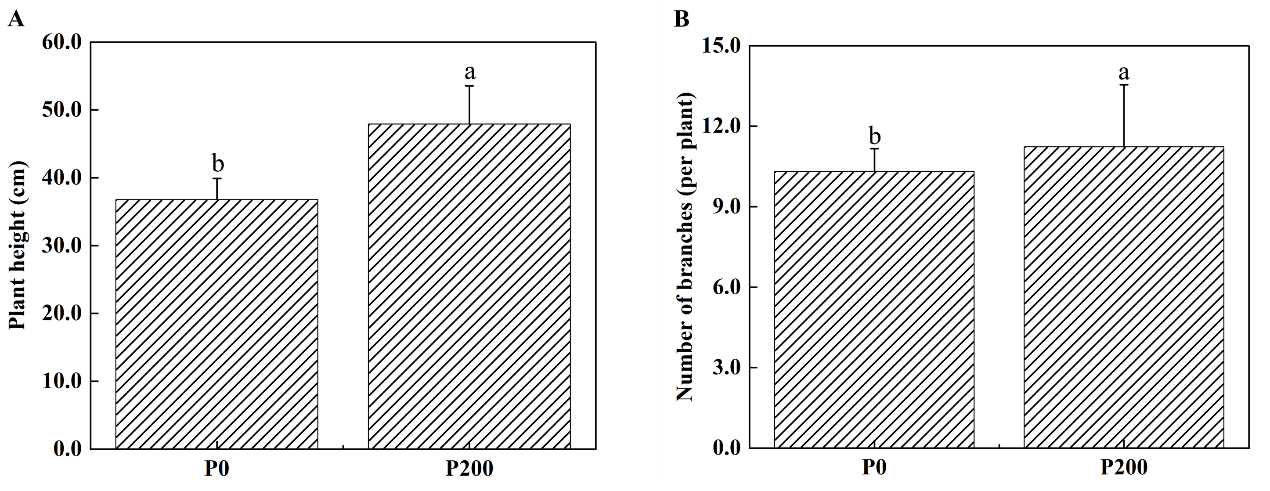


**Figure S3**. Alfalfa height **(A)**, Alfalfa branch number **(B)** in pot experiment. Each value is the mean (+SD) of five replicates. Different letters denote significant differences (*P*<0.05).


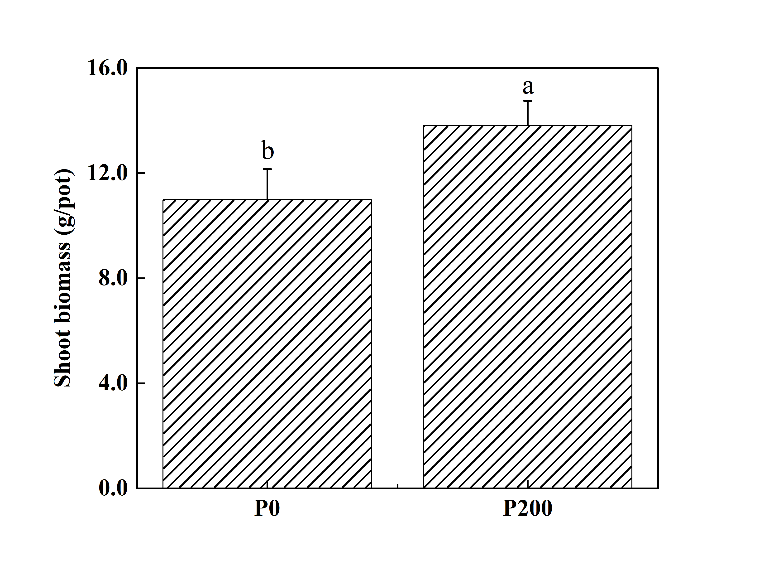


**Figure S4.** Alfalfa shoot biomass in pot experiment. Each value is the mean (+SD) of five replicates. Different letters denote significant differences (*P*<0.05).


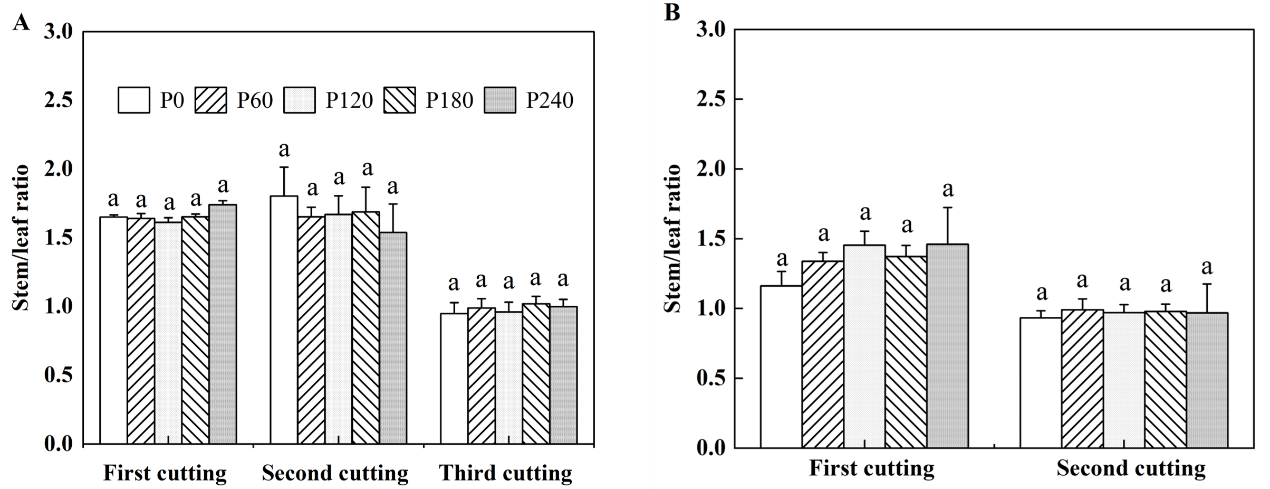


**Figure S5**. Alfalfa stem/leaf ratio in field experiment in 2021 **(A)** and 2022 **(B)**. Each value is the mean (+SE) of three replicates. Different letters denote significant differences (*P*<0.05).


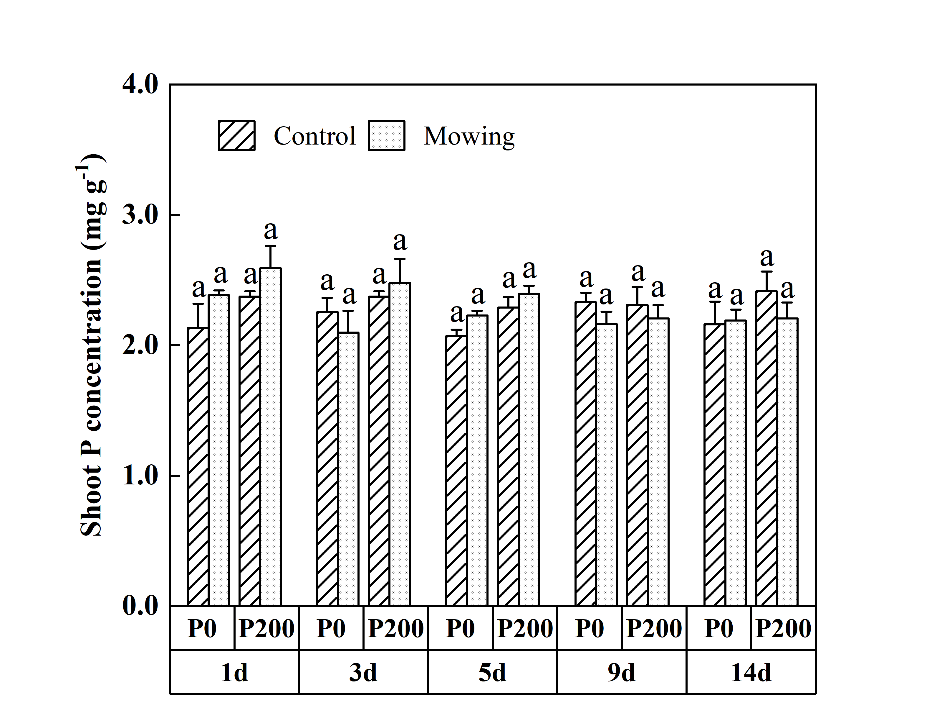


**Figure S6.** Alfalfa shoot P concentration in pot experiment. Each value is the mean (+SD) of three replicates. Different letters denote significant differences (*P*<0.05).
